# Menstrual cycle phase and early periorbital edema and ecchymosis after rhinoplasty: an observational study

**DOI:** 10.3389/fsurg.2026.1943506

**Published:** 2026-10-08

**Authors:** Yunus Emre Topan, Alper Tabaru, Ramazan Yavuz, Sedat Rüzgar

**Affiliations:** 1 Department of Otorhinolaryngology, Faculty of Medicine, Uşak University, Uşak, Türkiye; 2 Department of Otorhinolaryngology, University of Health Sciences, Istanbul, Türkiye

**Keywords:** ecchymosis, edema, estradiol, menstrual cycle, postoperative outcomes, progesterone, rhinoplasty

## Abstract

**Background:**

Whether estimated menstrual cycle phase affects postoperative recovery after rhinoplasty remains unclear. Although previous rhinoplasty research has addressed intraoperative bleeding, its relationship with visible early postoperative outcomes such as periorbital edema and ecchymosis has not been well characterized.

**Methods:**

This prospective observational cohort study included 72 regularly menstruating women who underwent primary open rhinoplasty between February 1 and December 1, 2025. All procedures were performed by the same senior surgeon using a standardized piezoelectric technique. Menstrual phase was estimated from the last menstrual period and usual cycle length using predefined calendar-based criteria. Preoperative serum estradiol and progesterone were measured as biochemical characteristics and were not used to determine phase assignment. Lower eyelid edema, upper eyelid edema, lower eyelid ecchymosis, and upper eyelid ecchymosis were scored separately on postoperative days 1 and 3 using a 0–4 ordinal scale by an independent evaluator blinded to menstrual phase and hormone values. Between-group comparisons were performed using the Kruskal–Wallis test with Dunn–Bonferroni *post hoc* analysis. Exploratory cumulative-logit mixed-effects models accounted for repeated assessments and adjusted for postoperative day, age, operative duration, and septal correction.

**Results:**

The mean age was 25.75 ± 5.46 years. Calendar-based classification identified 28 patients in the estimated follicular group, 8 in the estimated periovulatory group, and 36 in the estimated luteal group. Estradiol levels were highest in the periovulatory phase, whereas progesterone levels were highest in the luteal phase. Significant between-phase differences were observed for all edema and ecchymosis outcomes on both postoperative days. The follicular phase consistently showed the lowest scores. Although no statistically significant difference was detected between the periovulatory and luteal phases, the small periovulatory subgroup precludes interpreting this finding as evidence of equivalence. Adjusted analyses generally supported these associations, although the periovulatory–follicular contrast for upper eyelid edema was imprecise and not statistically significant (aOR 4.80, 95% CI 0.96–23.95).

**Conclusion:**

Estimated menstrual cycle phase was associated with early postoperative periorbital edema and ecchymosis, with generally lower scores in the follicular group. These exploratory findings are hypothesis-generating and do not establish causality or justify routine modification of rhinoplasty scheduling based solely on menstrual phase.

## Introduction

Rhinoplasty is one of the most commonly performed facial plastic procedures (1), and early postoperative recovery is strongly influenced by the degree of periorbital ecchymosis and edema (2). Although these findings are usually self-limited, they are important because they affect social downtime, patient anxiety, satisfaction with early recovery, and the perceived quality of surgery (3). Surgeons have therefore investigated multiple strategies to reduce postoperative bruising and swelling, including atraumatic osteotomy techniques, cold application, perioperative corticosteroids, controlled hypotension, local vasoconstrictors, and more recently piezoelectric osteotomy systems (4, 5).

Periorbital ecchymosis and edema after rhinoplasty are mainly attributed to bony and soft-tissue trauma during osteotomy and dissection, with subsequent vascular injury, inflammatory fluid accumulation, and lymphatic impairment (3, 6). Traditional osteotomies performed with osteotomes and mallets may produce less predictable fracture lines and adjacent mucosal or soft-tissue trauma (7). Piezoelectric surgery was introduced to allow more controlled cutting and sculpting of mineralized tissue under direct visualization. By using ultrasonic microvibrations, piezoelectric instruments selectively cut bone while theoretically reducing injury to surrounding soft tissue (8). For this reason, piezoelectric rhinoplasty has gained popularity, particularly in patients in whom minimizing postoperative edema and ecchymosis is a clinical priority. Nevertheless, even with standardized piezoelectric techniques, substantial interindividual variability remains (9).

The menstrual cycle is a potential biological contributor to this variability. Cyclical changes in estradiol and progesterone may influence vascular tone, endothelial function, connective tissue behavior, and hemostatic variables (10, 11). However, the clinical relevance of these changes for surgical outcomes is controversial. Some studies have found menstrual-cycle-related differences in perioperative bleeding (12), while others have not (13, 14). This inconsistency may reflect differences in surgical site, tissue hormone sensitivity, endpoint definition, and study design, suggesting that menstrual phase should not be regarded as a universal risk factor across all operations but rather as a potentially tissue-specific modifier.

The nose is a particularly relevant anatomical site for this question because the nasal mucosa may respond to cyclical hormonal changes. Previous studies of nasal physiology have produced mixed findings: some investigators did not confirm a consistent relationship between menstrual cycle phase and nasal congestion, whereas others reported cycle-related changes in nasal airflow, nasal resistance, decongestive response, or mucosal reactivity around periods of increased estrogen exposure (15, 16). These findings suggest that hormonal effects may not always manifest as baseline nasal obstruction alone, but may also involve altered mucosal or vascular responsiveness to local stimulation (17, 18).

Rhinoplasty-specific evidence on the relationship between menstrual cycle phase and perioperative outcomes remains limited. Although one previous study reported cycle-related differences in intraoperative blood loss during rhinoplasty, visible postoperative outcomes such as periorbital ecchymosis and edema have not been sufficiently characterized in this context (12). Therefore, the present study aimed to evaluate whether estimated menstrual cycle phase is associated with early postoperative periorbital ecchymosis and edema after rhinoplasty. To reduce surgical variability, all operations were performed by the same surgeon using a standardized piezoelectric technique. To reduce assessment bias, postoperative scoring was performed by an evaluator who did not participate in the operations. We hypothesized that patients in the estimated follicular group would have lower postoperative ecchymosis and edema scores than those in the estimated periovulatory or luteal groups.

## Materials and methods

### Study design and ethical approval

This single-center prospective observational cohort study was conducted at a tertiary referral center and included women of reproductive age who underwent primary open rhinoplasty between February 1 and December 1, 2025. Participants were enrolled preoperatively and followed through postoperative day 3. Ethical approval was obtained from the Uşak University Clinical Research Ethics Committee on January 12, 2025, with protocol code 018. The study was conducted in accordance with the principles of the Declaration of Helsinki, and written informed consent was obtained from all participants before enrollment. Reporting was guided by the Strengthening the Reporting of Observational Studies in Epidemiology (STROBE) recommendations for cohort studies.

### Participants

Consecutive women of reproductive age scheduled for primary open rhinoplasty during the study period were assessed for eligibility. Eligible patients reported regular menstrual cycles and had sufficient preoperative menstrual history to permit calendar-based phase estimation. All eligible patients were invited to participate irrespective of their estimated menstrual phase.

Exclusion criteria were irregular menstrual cycles, pregnancy, lactation, menopause, known bleeding disorders, active systemic inflammatory disease, anticoagulant or antiplatelet use, and current or recent hormonal treatment that could alter the menstrual cycle. Women using oral contraceptives or other exogenous sex hormones were excluded. Written informed consent was obtained before enrollment, and postoperative assessments were scheduled for days 1 and 3.

No *a priori* sample-size or power calculation was performed. Enrollment was based on eligible, consenting patients recruited during the defined study period. Menstrual-phase group sizes were not predetermined or balanced, and surgery was not scheduled or rescheduled according to menstrual phase for study purposes. Group membership reflected each participant's estimated phase on the day of surgery.

### Menstrual phase classification and hormonal assessment

Menstrual cycle phase at surgery was estimated from preoperative menstrual history. The first day of full menstrual bleeding was designated cycle day 1. The date of the last menstrual period and the durations of the preceding three cycles were recorded. Regular cycles were defined according to self-reported predictable menstrual intervals without recent irregularity. Each participant's usual cycle length was calculated as the median duration of the preceding three cycles.

The estimated ovulation day was calculated as the usual cycle length minus 14 days. For this exploratory study, the estimated follicular phase extended from cycle day 1 to two days before estimated ovulation. The estimated periovulatory phase included the day before, the estimated day of, and the day after ovulation. The estimated luteal phase extended from two days after estimated ovulation to the end of that cycle. For a 28-day cycle, these definitions corresponded to days 1–12, days 13–15, and day 16 onward, respectively. These were operational calendar-based definitions rather than validated biological boundaries. Group membership was determined by the estimated phase at surgery and retained for the analyses of both postoperative assessment days.

Blood samples were obtained on the day of surgery before anesthesia induction, local anesthetic infiltration, or surgical manipulation. Serum estradiol and progesterone concentrations were measured using chemiluminescent immunoassay on an automated Atellica Solution platform according to the manufacturer's instructions and internal quality-control procedures.

Hormone concentrations were reported as biochemical characteristics of the calendar-defined groups. No fixed estradiol or progesterone thresholds were used to assign, confirm, or override menstrual phase classification. Participants retained their calendar-based classification irrespective of their hormone concentrations; no exclusions or reassignments were made solely because hormone values differed from the expected phase-related pattern.

Serial hormone measurements, LH-surge testing, follicular ultrasonography, and basal body temperature monitoring were not performed. Accordingly, the study categories represented estimated menstrual phases rather than confirmed ovulatory status.

### Surgical technique

All operations were performed under general anesthesia by the same senior surgeon using a standardized open rhinoplasty technique. Intraoperative blood-pressure management followed the same protocol, with a target mean arterial pressure of 65–75 mmHg. Following anesthesia induction, 10 mL of 1% lidocaine with epinephrine at a concentration of 1:100,000 was infiltrated into the nasal dorsum, lateral nasal walls, septal region, and planned osteotomy lines. A minimum interval of 10 min was allowed for vasoconstriction before incision. An inverted-V transcolumellar incision connected to bilateral marginal incisions was then made, and the nasal soft-tissue envelope was elevated in the sub-SMAS and subperichondrial-subperiosteal planes.

Septal correction was performed when necessary, with preservation of an adequate L-strut. Dorsal modification was performed according to the individual deformity. In all cases requiring bony vault correction, piezoelectric instruments were used for bony vault sculpting, dorsal refinement, and controlled medial and lateral osteotomies. Piezoelectric osteotomies were performed under direct visualization with continuous irrigation to reduce thermal injury. The bony vault was mobilized and repositioned in a controlled manner, and sharp irregularities were refined with piezoelectric rasps as needed. Tip surgery, including cephalic trimming, interdomal sutures, transdomal sutures, septal extension grafts, or columellar strut grafts, was performed according to patient anatomy. Hemostasis was achieved with conservative bipolar cautery where necessary. Internal silicone splints and an external nasal splint were applied at the end of surgery.

Standardization concerned the operative approach, dissection planes, and piezoelectric instrumentation. The extent of dorsal modification, osteotomy, soft-tissue dissection, and grafting was individualized according to each patient's anatomy and corrective requirements. Operative duration and whether septal correction was performed were available for between-group comparisons. However, detailed information on osteotomy configuration and extent, dissection extent, and graft type and number was not systematically captured in the study dataset, and no formal operative complexity score was recorded. Therefore, comparability of these detailed procedural characteristics across the estimated menstrual phase groups could not be established.

Menstrual phase was not used to modify the surgical plan in any patient. The operating surgeon was not involved in the postoperative assessment or scoring of periorbital edema and ecchymosis.

### Perioperative management

The same perioperative care protocol was applied irrespective of estimated menstrual cycle phase. All patients received a single intravenous dose of dexamethasone (8 mg) after anesthesia induction and before incision. No additional corticosteroid doses were prescribed postoperatively. Antibiotic prophylaxis consisted of cefazolin (2 g intravenously) administered within 60 min before incision; no routine postoperative antibiotic course was prescribed. Tranexamic acid was not administered.

Postoperative care instructions specified cold-pack application to the periorbital region for 15 min every hour while awake during the first 48 h. Cold packs were wrapped in a thin cloth and applied without direct pressure on the nasal dorsum. Patients were instructed to maintain approximately 30° head elevation when resting or sleeping during the first postoperative week. Oral paracetamol, 500–1,000 mg every 8 h as needed, with a maximum daily dose of 3 g, was prescribed for postoperative analgesia. Aspirin, nonsteroidal anti-inflammatory drugs, and additional topical or oral treatments intended to reduce edema or ecchymosis were not prescribed.

All patients received identical written instructions regarding medication use, cold application, head elevation, and avoidance of nose blowing, bending forward, and strenuous physical activity during the early postoperative period. These instructions were not modified according to estimated menstrual cycle phase. Adherence to postoperative instructions after discharge was not objectively monitored.

### Postoperative assessment

Postoperative outcomes were assessed during in-person clinical examinations on postoperative days 1 and 3. Four parameters were scored separately: lower eyelid edema, upper eyelid edema, lower eyelid ecchymosis, and upper eyelid ecchymosis. Each parameter was graded using a semiquantitative 0–4 ordinal scale with the study-specific descriptive criteria detailed below. Clinical grading approaches for post-rhinoplasty edema and ecchymosis have been described in the literature (2), and the use of 0–4 scoring has previously been reported by Sakallioğlu et al. (19). For edema, scores were defined as follows: 0, no visible swelling; 1, minimal swelling; 2, mild-to-moderate localized swelling; 3, marked swelling without complete eyelid closure; and 4, severe swelling with pronounced eyelid narrowing or near-closure. For ecchymosis, scores were defined as follows: 0, no discoloration; 1, faint or minimal discoloration; 2, mild-to-moderate localized ecchymosis; 3, marked ecchymosis involving most of the assessed eyelid region; and 4, severe confluent ecchymosis. The specific descriptive criteria used in the present study were not formally validated. The same grading definitions were applied at both time points.

All assessments were performed by the same independent evaluator during routine postoperative follow-up. Patients were evaluated in an upright seated position, and the periorbital region was inspected directly under standard clinical examination conditions. The evaluator did not participate in the operations, was not involved in surgical decision-making, and was blinded to estimated menstrual phase assignment and hormone values at the time of scoring. These procedures were intended to reduce observer bias and assessment variability. However, no duplicate ratings for reliability testing or independent ratings by a second evaluator were obtained. Therefore, neither intraobserver nor interobserver reliability was formally assessed.

The primary outcomes were between-phase differences in lower eyelid edema, upper eyelid edema, lower eyelid ecchymosis, and upper eyelid ecchymosis scores on postoperative days 1 and 3. Secondary outcomes were within-patient postoperative change scores for each parameter, calculated as the postoperative day 3 score minus the postoperative day 1 score; positive values indicated worsening over time.

### Statistical analysis

Edema and ecchymosis scores were ordinal variables and are therefore presented as median and interquartile range. Postoperative edema and ecchymosis scores were compared among the estimated follicular, periovulatory, and luteal groups using the Kruskal–Wallis test. When the global test was statistically significant, pairwise comparisons were performed using Dunn's *post hoc* test with Bonferroni correction. Within-patient changes between postoperative days 1 and 3 were evaluated separately within each estimated menstrual-phase group using the Wilcoxon signed-rank test. Holm correction was applied across all 12 within-group comparisons, comprising four outcomes in each of the three phase groups. Unadjusted and Holm-adjusted *p* values were reported, and statistical significance was assessed using the adjusted *p* values. These secondary within-group comparisons constituted a separate hypothesis family from the eight adjusted between-phase contrasts evaluated in the exploratory mixed-effects analyses.

All 72 enrolled participants had complete data for menstrual history, hormone measurements, age, operative duration, septal correction, and the four postoperative outcomes at both assessments. No participants were excluded after enrollment because of missing data, and no imputation was performed.

Exploratory adjusted analyses were performed using cumulative-logit mixed-effects models. A separate model was fitted for each of the four ordinal outcomes: lower eyelid edema, upper eyelid edema, lower eyelid ecchymosis, and upper eyelid ecchymosis. Each model included estimated menstrual phase, postoperative day, age, operative duration, and septal correction as fixed effects, with a patient-specific random intercept to account for repeated assessments. The follicular group was the reference category. Age and operative duration were entered as continuous variables, and septal correction was coded as present or absent. Covariates were selected on clinical grounds rather than according to their statistical significance in baseline comparisons.

To limit model complexity, phase-by-day interaction terms were not included; therefore, the models estimated a common phase association across postoperative days 1 and 3. Model convergence, potential sparse-data instability, and the proportional-odds assumption were examined before interpretation. Results were expressed as adjusted odds ratios (aORs) with pointwise 95% Wald confidence intervals. An aOR greater than 1 indicated higher odds of a more severe outcome category. Two-sided Wald *p* values were adjusted using the Holm procedure across the eight comparisons of the periovulatory and luteal groups with the follicular reference group.

Continuous descriptive variables are presented as mean ± standard deviation. Normality was assessed before group comparisons. Normally distributed continuous variables were compared using one-way analysis of variance, whereas non-normally distributed continuous variables were compared using the Kruskal–Wallis test. Categorical variables are presented as number and percentage and were compared using the chi-square test or Fisher's exact test, as appropriate. Variables that were identical across all groups were not statistically compared.

Descriptive and unadjusted analyses were performed using IBM SPSS Statistics version 22.0. Exploratory cumulative-logit mixed-effects models were fitted in R using the ordinal package. Statistical significance was assessed at a two-sided level of 0.05, using multiplicity-adjusted *p* values where specified.

## Results

During the recruitment period, 90 consecutive women were assessed for eligibility. Fourteen did not meet the eligibility criteria: eight reported irregular menstrual cycles and six were using exogenous sex hormones. Of the remaining 76 eligible women, four declined participation. Seventy-two women were therefore enrolled, with a mean age of 25.75 ± 5.39 years. Twenty-eight were classified in the estimated follicular phase, eight in the periovulatory phase, and 36 in the luteal phase. All enrolled participants completed the postoperative day 1 and day 3 assessments, and all 72 were included in the analyses.

Baseline demographic, hormonal, and perioperative characteristics are presented in Table 1.

**Table 1:** Baseline demographic, hormonal, and perioperative characteristics according to estimated menstrual cycle phase.

Variable	Total *n* = 72	Follicular group *n* = 28	Periovulatory group *n* = 8	Luteal group *n* = 36	*p* value
Age, years	25.75 ± 5.39	25.3 ± 5.1	26.1 ± 6.0	26.0 ± 5.6	0.842
Estradiol, pg/mL	–	108.24 ± 22.80	149.40 ± 43.50	77.80 ± 26.40	<0.001
Progesterone, ng/mL	–	1.83 ± 0.70	2.16 ± 2.09	4.00 ± 2.90	<0.001
Operative duration, min	119.6 ± 18.4	118.9 ± 17.6	121.3 ± 18.8	119.7 ± 19.2	0.948
Septal correction, *n* (%)	56 (77.8)	22 (78.6)	6 (75.0)	28 (77.8)	0.977

Mean operative duration was 118.9 ± 17.6, 121.3 ± 18.8, and 119.7 ± 19.2 min in the follicular, periovulatory, and luteal groups, respectively (*p* = 0.948). Septal correction was performed in 22/28 patients (78.6%), 6/8 patients (75.0%), and 28/36 patients (77.8%), respectively (*p* = 0.977). Neither variable showed a statistically significant between-group difference (Table 1).

In the main postoperative comparisons, significant between-group differences were observed for all assessed edema and ecchymosis outcomes on both postoperative day 1 and postoperative day 3. The follicular group consistently showed the lowest postoperative edema and ecchymosis scores, whereas the periovulatory and luteal groups showed higher scores. The distribution of postoperative scores according to menstrual cycle phase is illustrated in Figure 1, and the detailed median values, interquartile ranges, Kruskal–Wallis *p* values, and Dunn–Bonferroni *post hoc* comparisons are presented in Table 2.

**Figure 1:**
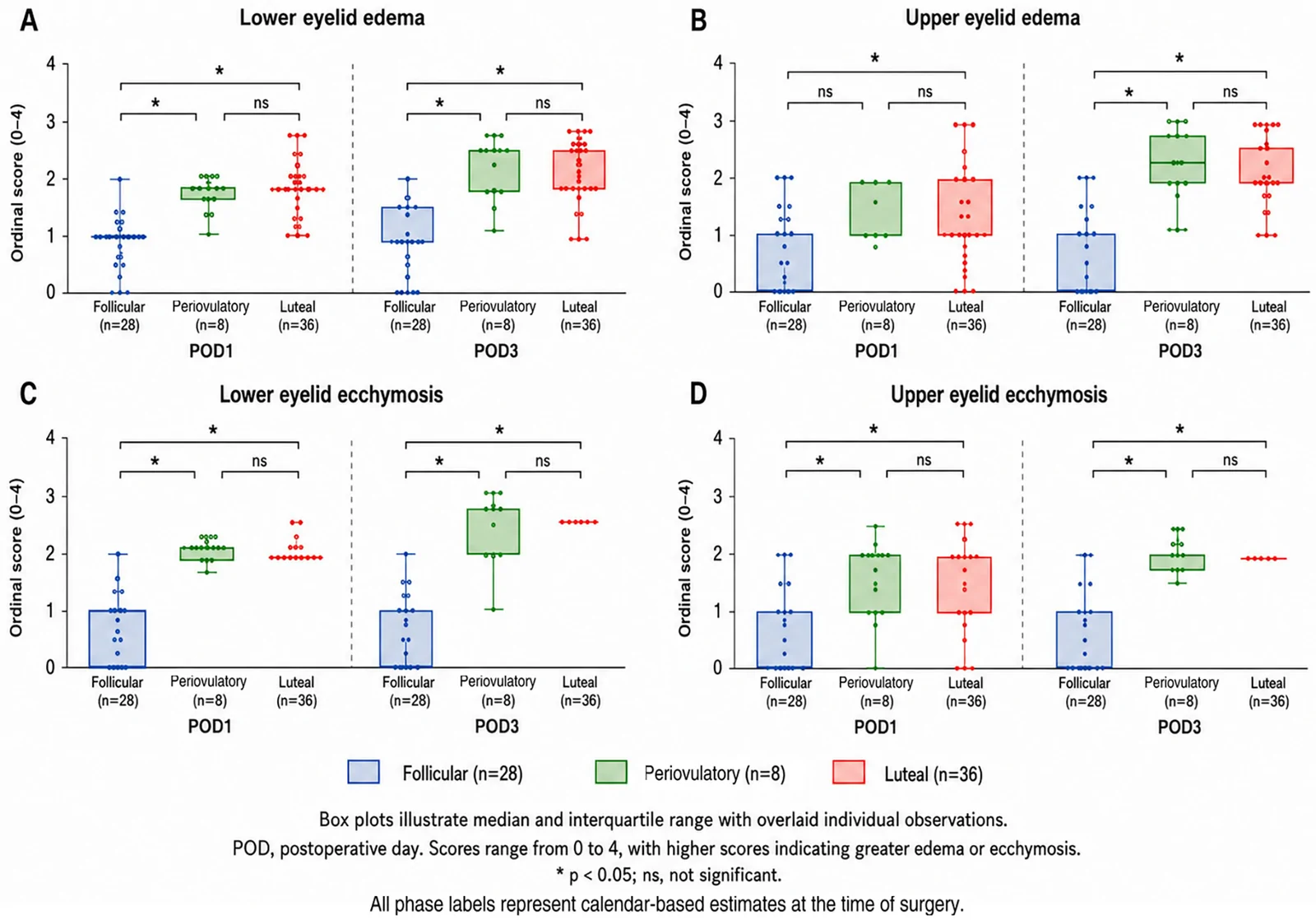
Early postoperative periorbital edema and ecchymosis according to estimated menstrual cycle phase. Panels show (A) lower eyelid edema, (B) upper eyelid edema, (C) lower eyelid ecchymosis, and (D) upper eyelid ecchymosis on postoperative days 1 and 3. Box plots illustrate the median and interquartile range, with individual observations overlaid. Scores range from 0 to 4, with higher scores indicating greater edema or ecchymosis. POD, postoperative day. *p < 0.05 after Dunn-Bonferroni correction; ns, not significant.

**Table 2:** Postoperative edema and ecchymosis scores according to estimated menstrual cycle phase.

Outcome	Time point	Follicular group *n* = 28	Periovulatory group *n* = 8	Luteal group *n* = 36	Kruskal–Wallis *p*	Dunn–Bonferroni *post hoc*
Lower eyelid edema	POD1	1.0 [1.0–1.0]	2.0 [1.8–2.0]	2.0 [2.0–2.0]	<0.001	F < PO, *p* = 0.019; F < L, *p*<0.001; PO vs. L, *p* = 1.000
Upper eyelid edema	POD1	1.0 [0.0–1.0]	1.0 [1.0–2.0]	1.0 [1.0–2.0]	<0.001	F vs. PO, *p* = 0.112; F < L, *p*<0.001; PO vs. L, *p* = 1.000
Lower eyelid ecchymosis	POD1	1.0 [0.0–1.0]	2.0 [1.8–2.0]	2.0 [2.0–2.0]	<0.001	F < PO, *p* = 0.001; F < L, *p*<0.001; PO vs. L, *p* = 1.000
Upper eyelid ecchymosis	POD1	0.0 [0.0–1.0]	2.0 [1.0–2.0]	2.0 [1.0–2.0]	<0.001	F < PO, *p* = 0.001; F < L, *p*<0.001; PO vs. L, *p* = 1.000
Lower eyelid edema	POD3	1.0 [1.0–2.0]	3.0 [2.0–3.0]	3.0 [2.0–3.0]	<0.001	F < PO, *p* = 0.001; F < L, *p*<0.001; PO vs. L, *p* = 1.000
Upper eyelid edema	POD3	1.0 [0.0–1.0]	2.5 [2.0–3.0]	2.0 [2.0–3.0]	<0.001	F < PO, *p*<0.001; F < L, *p*<0.001; PO vs. L, *p* = 1.000
Lower eyelid ecchymosis	POD3	1.0 [0.0–1.0]	3.0 [2.0–3.0]	3.0 [3.0–3.0]	<0.001	F < PO, *p*<0.001; F < L, *p*<0.001; PO vs. L, *p* = 1.000
Upper eyelid ecchymosis	POD3	0.0 [0.0–1.0]	2.0 [1.8–2.3]	2.0 [2.0–2.0]	<0.001	F < PO, *p*<0.001; F < L, *p*<0.001; PO vs. L, *p* = 1.000

Data are presented as median [interquartile range]. POD, postoperative day; F, follicular group; PO, periovulatory group; L, luteal group. Between-group comparisons were performed using the Kruskal–Wallis test. Pairwise comparisons were performed using Dunn's *post hoc* test with Bonferroni correction.

On postoperative day 1, lower eyelid edema was lower in the follicular group than in both the periovulatory and luteal groups. Upper eyelid edema was also lower in the follicular group than in the luteal group, whereas the difference between the follicular and periovulatory groups did not reach statistical significance. For lower and upper eyelid ecchymosis on postoperative day 1, the follicular group showed significantly lower scores than both the periovulatory and luteal groups. No significant difference was detected between the periovulatory and luteal groups for any postoperative day 1 outcome.

On postoperative day 3, the follicular group had significantly lower scores than both the periovulatory and luteal groups for all four outcomes in the unadjusted comparisons (Table 2). For both lower eyelid edema and lower eyelid ecchymosis, median scores were 1.0 in the follicular group and 3.0 in both other groups. Median upper eyelid edema scores were 1.0, 2.5, and 2.0 in the follicular, periovulatory, and luteal groups, respectively. Median upper eyelid ecchymosis scores were 0.0 in the follicular group and 2.0 in both other groups. Thus, descriptive differences between group medians relative to the follicular group ranged from 1.0 to 2.0 points on the 0–4 scale. No statistically significant periovulatory–luteal differences were detected in the day-specific comparisons.

Within-group changes from postoperative day 1 to day 3 are summarized in Table 3. After Holm correction across the 12 comparisons, the increase in lower eyelid edema remained statistically significant in the follicular group, whereas the other three follicular comparisons did not reach statistical significance. None of the four periovulatory comparisons remained statistically significant after adjustment. In the luteal group, increases in all four edema and ecchymosis outcomes remained statistically significant. Positive change scores indicated higher severity scores on postoperative day 3.

**Table 3:** Within-group changes in postoperative edema and ecchymosis scores from postoperative day 1 to day 3.

Menstrual phase	Outcome	POD1 median [IQR]	POD3 median [IQR]	Median change [IQR]	Wilcoxon *p*	Holm-adjusted *p*
Follicular group *n* = 28	Lower eyelid edema	1.0 [1.0–1.0]	1.0 [1.0–2.0]	0.0 [0.0–1.0]	0.003	0.024
Follicular group *n* = 28	Upper eyelid edema	1.0 [0.0–1.0]	1.0 [0.0–1.0]	0.0 [0.0–0.0]	0.041	0.123
Follicular group *n* = 28	Lower eyelid ecchymosis	1.0 [0.0–1.0]	1.0 [0.0–1.0]	0.0 [0.0–1.0]	0.018	0.090
Follicular group *n* = 28	Upper eyelid ecchymosis	0.0 [0.0–1.0]	0.0 [0.0–1.0]	0.0 [0.0–0.0]	0.317	0.317
Periovulatory group *n* = 8	Lower eyelid edema	2.0 [1.8–2.0]	3.0 [2.0–3.0]	1.0 [0.0–1.0]	0.026	0.104
Periovulatory group *n* = 8	Upper eyelid edema	1.0 [1.0–2.0]	2.5 [2.0–3.0]	1.0 [0.5–1.0]	0.012	0.077
Periovulatory group *n* = 8	Lower eyelid ecchymosis	2.0 [1.8–2.0]	3.0 [2.0–3.0]	1.0 [1.0–1.0]	0.011	0.077
Periovulatory group *n* = 8	Upper eyelid ecchymosis	2.0 [1.0–2.0]	2.0 [1.8–2.3]	0.5 [0.0–1.0]	0.046	0.123
Luteal group *n* = 36	Lower eyelid edema	2.0 [2.0–2.0]	3.0 [2.0–3.0]	1.0 [1.0–1.0]	<0.001	<0.05
Luteal group *n* = 36	Upper eyelid edema	1.0 [1.0–2.0]	2.0 [2.0–3.0]	1.0 [0.0–1.0]	<0.001	<0.05
Luteal group *n* = 36	Lower eyelid ecchymosis	2.0 [2.0–2.0]	3.0 [3.0–3.0]	1.0 [1.0–1.0]	<0.001	<0.05
Luteal group *n* = 36	Upper eyelid ecchymosis	2.0 [1.0–2.0]	2.0 [2.0–2.0]	1.0 [0.0–1.0]	<0.001	<0.05

Data are presented as median [interquartile range]. Median change was calculated as POD3 score - POD1 score. Positive values indicate worsening from postoperative day 1 to postoperative day 3. Within-group comparisons were performed using the Wilcoxon signed-rank test. Holm correction was applied across the 12 within-group comparisons involving four outcomes in three menstrual-phase groups. Statistical significance was assessed using Holm-adjusted *p* values below 0.05.

POD, postoperative day; IQR, interquartile range.

Exploratory adjusted analyses are presented in Table 4. After accounting for postoperative day, age, operative duration, septal correction, and within-patient repeated measurements, the luteal group had higher odds of more severe scores than the follicular group for all four outcomes, with adjusted odds ratios ranging from 5.60 to 24.00 (all Holm-adjusted *p* < 0.001). The periovulatory group also had higher odds of more severe lower eyelid edema (aOR 10.50, 95% CI 2.46–44.78; adjusted *p* = 0.004), lower eyelid ecchymosis (aOR 18.00, 95% CI 3.75–86.34; adjusted *p* = 0.001), and upper eyelid ecchymosis (aOR 9.50, 95% CI 2.14–42.13; adjusted *p* = 0.006). For upper eyelid edema, the periovulatory–follicular estimate was imprecise and did not reach statistical significance (aOR 4.80, 95% CI 0.96–23.95; adjusted *p* = 0.056). These estimates describe a common association across the two postoperative assessments rather than separate day-specific effects.

**Table 4:** Exploratory adjusted associations between estimated menstrual cycle phase and postoperative edema and ecchymosis.

Outcome	Phase comparison	Adjusted OR	95% CI	Holm-adjusted *p*
Lower eyelid edema	Periovulatory vs. follicular	10.50	2.46–44.78	0.004
Lower eyelid edema	Luteal vs. follicular	12.00	4.78–30.15	<0.001
Upper eyelid edema	Periovulatory vs. follicular	4.80	0.96–23.95	0.056
Upper eyelid edema	Luteal vs. follicular	5.60	2.36–13.27	<0.001
Lower eyelid ecchymosis	Periovulatory vs. follicular	18.00	3.75–86.34	0.001
Lower eyelid ecchymosis	Luteal vs. follicular	24.00	8.66–66.50	<0.001
Upper eyelid ecchymosis	Periovulatory vs. follicular	9.50	2.14–42.13	0.006
Upper eyelid ecchymosis	Luteal vs. follicular	11.50	4.49–29.46	<0.001

Results are based on separate cumulative-logit mixed-effects models for the four outcomes, each including estimated menstrual phase, postoperative day, age, operative duration, and septal correction as fixed effects, with a patient-specific random intercept. Each model included 72 patients contributing 144 observations. Estimates represent the common phase association across postoperative days 1 and 3. An adjusted OR >1 indicates higher odds of a more severe score category. Confidence intervals are pointwise and were not adjusted for multiplicity; *p* values were Holm-adjusted across the eight phase contrasts.

OR, odds ratio; CI, confidence interval.

## Discussion

The main finding of this study was that patients in the estimated follicular group had lower early postoperative periorbital edema and ecchymosis scores than those in the estimated periovulatory or luteal groups. This pattern was evident on both postoperative day 1 and postoperative day 3. After correction for multiple within-group comparisons, evidence of worsening from postoperative day 1 to day 3 was retained for lower eyelid edema in the follicular group and for all four outcomes in the luteal group. Nonsignificant findings in the remaining comparisons should not be interpreted as evidence of unchanged postoperative severity. Furthermore, differences in statistical significance between groups do not demonstrate differences in their temporal trajectories; phase-by-day interactions were not evaluated in the adjusted models.

These findings describe differences in early clinician-rated postoperative appearance, although their implications for patient-perceived recovery remain uncertain. The exploratory adjusted analyses generally supported the pattern observed in the unadjusted comparisons, with higher postoperative severity scores in the luteal group and, for most outcomes, the periovulatory group relative to the follicular group. However, the confidence intervals were wide, particularly for the periovulatory contrasts, indicating substantial uncertainty regarding the magnitude of these associations. The adjusted odds ratios describe differences in the odds of occupying higher severity categories and should not be interpreted as proportional increases in swelling or bruising. Adjustment for the available covariates does not establish causality or demonstrate that changing the timing of surgery would improve postoperative recovery.

The present findings fit reasonably well within the existing rhinoplasty literature, although they extend it in a different direction. Findikcioglu et al. previously reported that intraoperative blood loss during rhinoplasty was higher in the periovulatory phase than in the perimenstrual phase (12). Unlike that study, the present study did not directly measure intraoperative blood loss; instead, it evaluated visible postoperative periorbital ecchymosis and edema. The observation that the follicular group showed the mildest postoperative periorbital soft-tissue response is therefore directionally compatible with previous evidence suggesting that menstrual-cycle phase may influence perioperative bleeding, vascular/endothelial function, hemostatic variables, and nasal mucosal physiology (10, 16, 20). However, intraoperative bleeding and postoperative ecchymosis or edema are related but not interchangeable endpoints. The lack of a clear periovulatory–luteal separation in the present study may be explained by differences in outcome definition, menstrual phase classification, timing of postoperative assessment, and the relatively small number of patients in the periovulatory subgroup.

Recent physiologic literature may help explain why a follicular advantage is biologically plausible. Contemporary rhinology reviews indicate that sex-related hormonal variation may influence sinonasal physiology, although the available evidence remains heterogeneous and not fully consistent (17, 21). More recent menstrual-cycle studies have reported phase-dependent differences in nasal resistance, nasal patency, subjective obstruction scores, and decongestive response (15, 16). Taken together, these findings suggest that menstrual-cycle-related hormonal changes may not produce a uniform baseline obstructive pattern, but may alter nasal mucosal or vascular responsiveness (15–17). Rhinoplasty may represent a clinically relevant form of local provocation, because osteotomy, soft-tissue elevation, mucoperichondrial manipulation, and periosteal disruption create local vascular and inflammatory stimuli that may contribute to visible bruising and swelling (3). The present findings indicate an association between calendar-defined menstrual phase groups and early postoperative periorbital appearance; they do not establish a causal hormonal mechanism.

The broader operative literature supports a tissue- and endpoint-specific interpretation. Studies in abdominoplasty, laparoscopic myomectomy, vitreoretinal surgery, and posterior spinal fusion have not demonstrated a clinically meaningful effect of menstrual timing on perioperative blood loss (14, 22–24). In contrast, positive findings have been reported in LEEP and rhinoplasty, suggesting that phase-related effects may become more apparent in selected operative settings, particularly when the surgical field is mucosal, highly vascular, or sensitive to local bleeding and postoperative extravasation (12, 25). Taken together, these data do not support menstrual phase as a universal bleeding risk factor across all operations. Rather, they suggest that its clinical relevance may depend on the operative site, local tissue biology, outcome definition, and study design.

Menstrual phase in this study was estimated from menstrual history and usual cycle length, while preoperative estradiol and progesterone concentrations were reported separately as biochemical characteristics. Calendar-based methods are susceptible to phase misclassification because menstrual timing and the relationship between cycle day and ovulation vary between individuals and between cycles (26, 27). Although mean estradiol concentrations were highest in the periovulatory group and mean progesterone concentrations were highest in the luteal group, these group-level patterns do not validate each participant's phase assignment. The hormonal measurements therefore describe the endocrine characteristics of the study groups without establishing that all participants were correctly classified.

From a clinical perspective, the observed score differences should be interpreted using the study-specific grading definitions. For lower eyelid edema and ecchymosis, the postoperative day 3 median scores of 1 in the follicular group and 3 in the other groups corresponded to minimal swelling or faint discoloration versus marked swelling or ecchymosis. These differences indicate separation in clinician-rated severity categories. However, the scale is ordinal, and a two-point difference should not be interpreted as a fixed quantitative reduction in swelling volume or bruised area. A minimal clinically important difference was not established for the specific grading criteria used in this study.

The study did not assess whether lower scores translated into faster resolution of symptoms, earlier return to usual social activities, or greater patient satisfaction. The magnitude of any patient-important benefit therefore remains uncertain. Moreover, the observational design cannot establish that moving an individual patient's surgery to the follicular phase would improve postoperative recovery. Given the estimated phase classification, small periovulatory subgroup, and potential residual confounding, these findings should be regarded as hypothesis-generating. They do not currently justify routine modification of surgical scheduling based solely on menstrual phase. Larger prospective studies incorporating more robust phase assessment, detailed evaluation of perioperative confounders, and validated patient-reported outcomes are needed to assess the reproducibility and clinical relevance of these associations.

### Limitations

This study has several limitations. First, no *a priori* sample-size calculation was performed, and the sample was relatively small and unevenly distributed across menstrual phases. The periovulatory subgroup comprised only eight patients, limiting the ability to detect between-phase differences and the precision and stability of estimates, including those from adjusted analyses. The absence of statistically significant periovulatory–luteal differences should therefore not be interpreted as evidence of equivalence.

Second, menstrual phase was estimated from self-reported menstrual history and calendar-based criteria without confirmation of ovulation. Recall error and individual variation in ovulation timing and luteal-phase length may have resulted in phase misclassification. Single preoperative estradiol and progesterone measurements provided descriptive endocrine information but were not used to confirm or revise phase assignment. The direction and magnitude of any resulting bias remain uncertain.

Third, although all procedures were performed by a single surgeon using a common operative approach and piezoelectric instrumentation, procedural extent was individualized. Exploratory models adjusted for age, operative duration, and septal correction; however, the latter two variables are incomplete indicators of surgical complexity, and the absence of statistically significant between-group differences in these measures does not establish equivalent procedural extent. Osteotomy extent, soft-tissue dissection, and grafting characteristics were not systematically quantified or compared and were unavailable for adjustment. Residual confounding related to procedural tissue trauma, nasal skin thickness, and individual inflammatory responses therefore cannot be excluded. Perioperative prescriptions and postoperative instructions were standardized, but adherence to cold application, head elevation, and medication instructions after discharge was not objectively monitored. Differences in adherence may also have influenced the outcomes. Furthermore, the models assumed a common association between menstrual phase and outcome scores across postoperative days 1 and 3; a phase-by-day interaction was not evaluated.

Fourth, postoperative outcomes were assessed using a semiquantitative ordinal scale with study-specific descriptive criteria that had not undergone formal validation. Although a blinded evaluator applied consistent grading definitions and examination procedures, neither intraobserver nor interobserver reliability was quantified. Score reproducibility therefore remains uncertain. Subjective interpretation and measurement error may have influenced the findings, with an unknown direction and magnitude of bias. Objective volumetric measurements of edema and digital quantification of ecchymosis were not obtained. A minimal clinically important difference was also not established for the grading criteria. Patient-reported recovery, satisfaction, and time to return to usual activities were not assessed, limiting interpretation of the observed score differences in terms of patient-important benefit.

Finally, although consecutive recruitment was used to reduce discretionary selection, participation was restricted to eligible, consenting women, and selection bias cannot be excluded. The single-center, single-surgeon setting and restriction to regularly menstruating women undergoing primary open piezoelectric rhinoplasty limit generalizability to other populations and surgical settings. Follow-up was confined to postoperative days 1 and 3, precluding evaluation of longer-term recovery, aesthetic outcomes, and revision rates.

## Conclusion

Estimated menstrual cycle phase was associated with early clinician-rated periorbital edema and ecchymosis after rhinoplasty, with generally lower scores in the follicular group. However, the observational design, uncertainty in phase classification, small periovulatory subgroup, and potential residual confounding limit interpretation. These findings are hypothesis-generating and do not demonstrate that scheduling surgery during the follicular phase improves recovery. Routine modification of rhinoplasty scheduling based solely on menstrual phase is therefore not justified by the present findings. Further prospective investigation is needed to establish the reproducibility and patient-important clinical relevance of the observed associations.

## Data Availability

The de-identified data supporting the conclusions of this article will be made available by the corresponding author upon reasonable request
